# Virtual Reality Simulation for the Acquisition and Retention of Electrocardiogram Interpretation Skills: A Randomized Controlled Trial Among Undergraduate Medical Students

**DOI:** 10.7759/cureus.62170

**Published:** 2024-06-11

**Authors:** Nayyar Iqbal, Ravichandran Kandasamy, Johnson O, Balasundaram R, Karthika Jyothish

**Affiliations:** 1 General Medicine, Pondicherry Institute of Medical Sciences, Pondicherry, IND; 2 Biostatistics, Pondicherry Institute of Medical Sciences, Pondicherry, IND; 3 Physiology, Pondicherry Institute of Medical Sciences, Pondicherry, IND

**Keywords:** traditional learning, randomized controlled trial (rct), medical education, electrocardiography (ecg), immersive virtual reality simulation

## Abstract

Introduction

The electrocardiogram (ECG) is one of the most important tools in diagnosing cardiac abnormalities, particularly arrhythmias and myocardial infarction. It is one of the certifiable competencies for final-year medical undergraduate students. We determined virtual reality's effectiveness in acquiring and retaining ECG interpretation skills among medical students compared to traditional teaching.

Methods

One hundred and forty students were randomized into two groups. Seventy-one students (immersion group) were trained using virtual reality simulation to acquire and retain interpretation skills of normal and abnormal ECG. Sixty-nine students (traditional group) were trained in the classroom using chalk and board. The primary outcome of change in acquiring knowledge of the interpretation of ECG was determined by comparing pre and post-test scores. The secondary outcome of retention of knowledge was determined by comparing pre-test and second post-test scores conducted after eight weeks of intervention. The p-value of <0.05 was considered significant.

Results

Out of 140 students, 50 (35.7%) were males and 90 (64.3%) were female. The mean age of the students was 22.1 (SD 1.1), with 69.3% of them between the ages of 21 and 22 years. Mean pre-test scores for the interpretation of normal ECG among immersion and traditional groups were 9.8 (SD 8.4) and 8.3 (SD 7.5), respectively, and post-test scores for the acquisition of knowledge were 24.3 (SD 5.5) and 24.8 (SD 6.3), respectively. The post-test scores for retention skills were 25.3 (SD 5.6) and 20.7 (SD 6.9) respectively (p<0.001). The mean pre-test scores for the interpretation of abnormal ECG of both groups were 7.0 (SD 6) and 8.3 (SD 6.6), respectively. Mean post-test scores for acquiring knowledge to interpret abnormal ECG were 23.5 (SD 6.2) and 17.7 (SD 9), respectively (p<0.001), and mean post-test scores for retention of interpretation skills of abnormal ECG were 19.2 (SD - 6.9) and 13.3 (SD 10.2) respectively (p=0.001). The pairwise comparison of the immersion group indicates that all the combinations that changed in score from the pre to post-intervention time points, from pre-to-retention time, and from the post-to-retention time were significant (p<0.001).

Conclusion

Virtual reality teaching had a better impact on acquiring and retaining the skill for interpreting normal and abnormal electrocardiograms.

## Introduction

An electrocardiogram (ECG) is one of the main tools for diagnosing heart conditions like arrhythmias, myocardial infarction, electrolyte abnormalities like hypokalemia or hyperkalemia, and pericardial diseases. ECG training is an integral part of the undergraduate medical curriculum. It is one of the competencies taught in the first year of medical school by a department of physiology. Studies have shown that medical graduates across the globe are not competent in interpreting the ECG.

Lever et al. from Auckland City Hospital in New Zealand conducted a study among final-year medical students, where all students were given various ECGs to interpret. The study found that students could not interpret most of the abnormalities. Students also rated their abilities below satisfactory in interpreting the ECG [1]. Amini et al*.* from Ardabil University of Medical Sciences, Iran, conducted a cross-sectional study among 323 medical students and found that a large number of the participants were unable to differentiate normal ECG from abnormal ECG [2].

Traditional ECG training has focused on the delivery of the content in didactic form with a case-based approach and combined with demonstrations in practical classes. Studies have also shown that blended learning involving technology like web-based tutorials was effective in achieving better competency levels [3]. The National Medical Commission of India, in a competency-based curriculum, has made ECG one of the certifiable competencies for final-year undergraduate medical students. We, therefore, designed a new approach to train undergraduates in the interpretation of ECG using virtual reality to determine its effectiveness in acquiring and retaining the skills to interpret ECG as compared to the traditional approach.

## Materials and methods

In this randomized controlled study, 140 final-year medical undergraduate students were assigned randomly using a computer-generated sequence of random numbers to two groups - the immersion group and the traditional teaching group. This study was conducted after getting approval from the Institutional Ethics Committee of Pondicherry Institute of Medical Sciences (IEC: RC/2023/77). Oral informed consent was obtained from all participants before their inclusion in the study.

**Figure 1:**
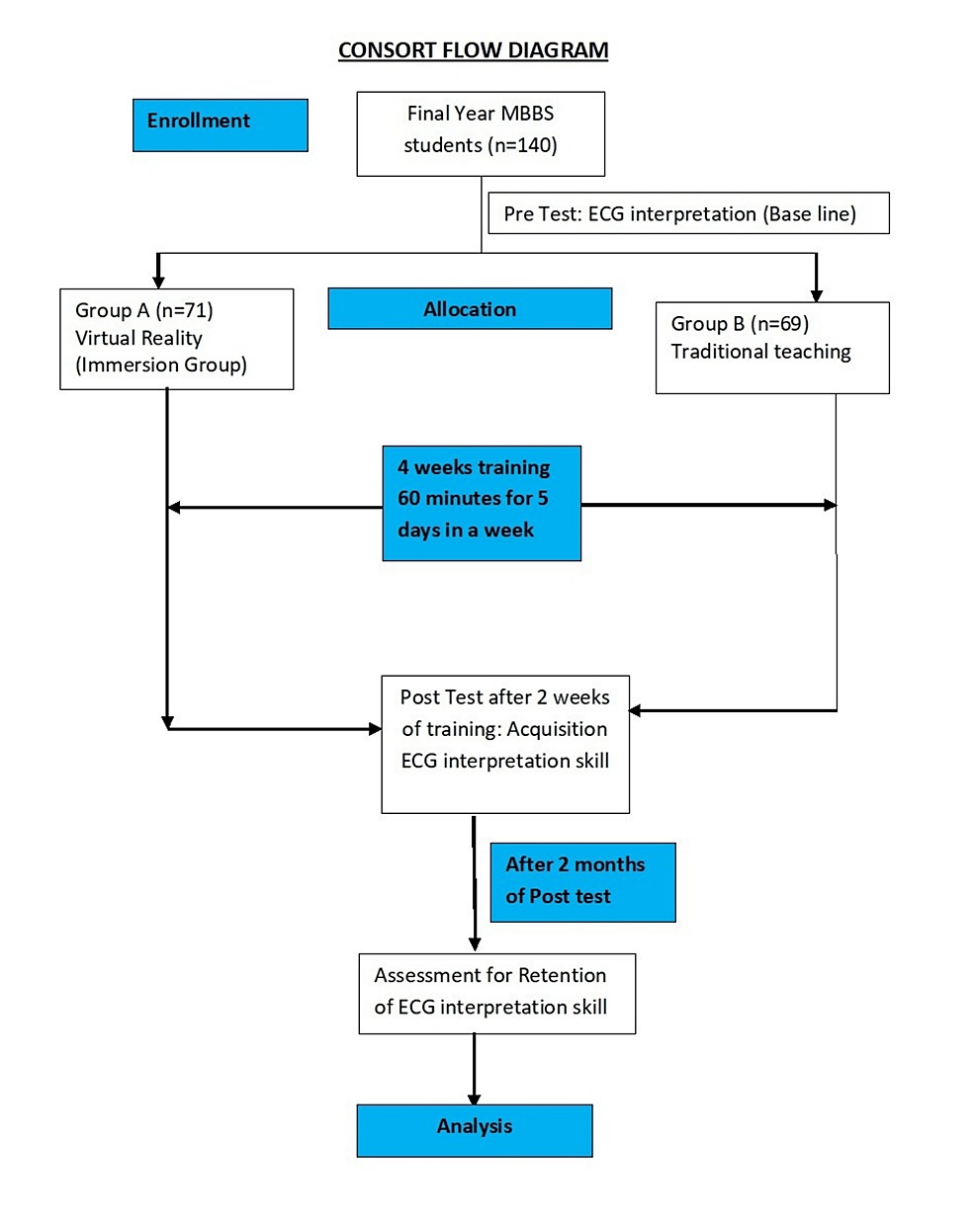
CONSORT flow diagram of participant recruitment, intervention, and results CONSORT - Consolidated Standards of Reporting Trials

Both the groups completed the pre-test by interpreting one normal and one abnormal ECG at baseline. The immersion group was trained using virtual reality simulation, and the traditional teaching group was trained by didactic lecture. Both groups were trained for one hour daily, five days a week for four weeks. They were trained to interpret normal ECG and ECG of various types of myocardial infarctions and atrial fibrillation.

Two weeks after the completion of training both groups took a post-test to assess the acquisition of knowledge. Two months later, the retention of knowledge was assessed by a retention post-test.

The brief description of virtual reality (VR) simulation is as follows. Each student was assigned a workstation with a virtual reality simulation, where each one of them had to wear a VR headset and access the virtual room with a patient. They did an ECG of the patient by virtually placing the leads and recording the ECG. After this, they were asked to interpret it. Students were allowed to select the module they wanted to learn - normal or abnormal ECG. Students were also guided by the faculty for any explanation required by them. Although a faculty was there to help them if required, VR simulation was a method of self-directed learning. They had an opportunity to access the module multiple times. In the traditional teaching group, the students were taught by chalk and board, and ECG were projected using an liquid-crystal display (LCD) projector. Then, they were asked to interpret the ECG. In the traditional teaching group, students were advised to refer to textbooks for a better understanding of ECG.

In pre and post-tests, students were asked to interpret the ECG regarding rhythm, rate, PR interval, axis, and final diagnosis. Each parameter was graded with five marks each and ten marks for correct diagnosis. The assessment of the pre and post-test was done by the blinded faculty who were not involved in the training of students.

Statistical analysis

Continuous data were expressed as mean along with standard deviation, while categorical variables were presented as numbers and percentages. A two-way mixed ANOVA, with one between-subjects factor and one within-subjects factor, was conducted to investigate whether the score in interpreting ECG over time differed by lecture type. The univariate repeated-measures tests and their sphericity corrections were examined. A post hoc test of pair-wise comparison was done using the Bonferroni method. A p-value (two-sided) was lowered below 0.05 for multiple comparison and considered indicative of statistical significance. The analysis was performed using SPSS statistics version 29 (IBM Inc., Armonk, New York).

## Results

Out of 140 students, 50 (35.7%) were males. The mean age of the students was 22.1 (SD 1.1), with 69.3% of them between the ages of 21 and 22 years. ECG classes in physiology were attended by 133 (95.0%) students, out of which only 78 (58.6%) remember the basics of ECG. There was no significant difference between the groups concerning gender, attending ECG class in the first year, and remembering the basics of ECG. The major baseline characteristics of students in the two groups are summarized in Table 1. 

**Table 1:** Baseline characteristics of study participants * details based on those who attended ECG class in the first year

Characteristics	Immersion group (n=71)	Traditional group (n=69)
Male	26 (36.6%)	24 (34.8%)
Female	45 (63.4%)	45 (65.2%)
Attended ECG class in the first year of MBBS	69 (97.2%)	64 (92.8%)
Remembered the basics of ECG*		
Yes	42 (60.9%)	36 (56.3%)
No	27 (39.1%)	28 (43.8%)

**Figure 2:**
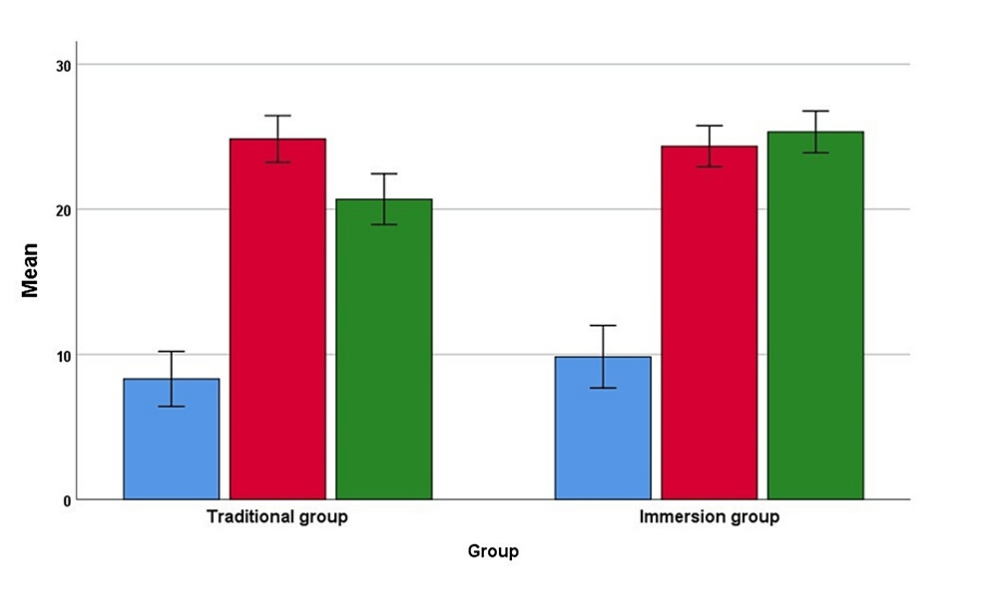
Scores of baseline (pre-test), after two weeks of completion of the intervention (post-test) and eight weeks of completion of the intervention (retention post-test) by groups for normal ECG Error bar 95% confidence interval Blue bar - mean pre-test score; red bar - post-test score; green bar - retention post-test

**Figure 3:**
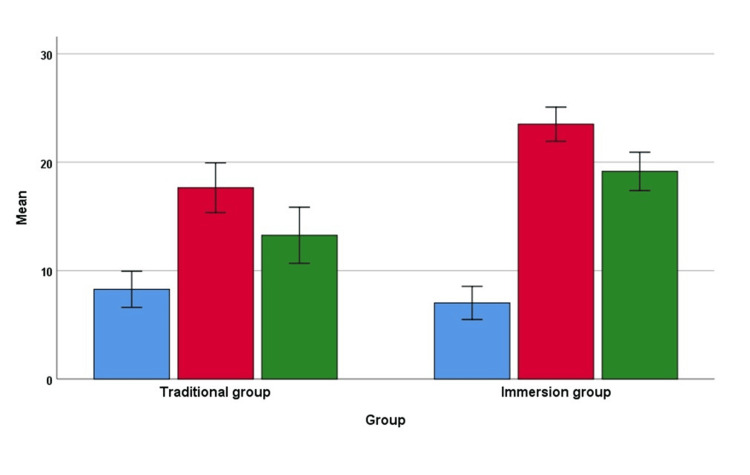
Scores of baseline (pre-test), after two weeks of completion of the intervention (post-test) and eight weeks of completion of the intervention (retention post-test) by groups for abnormal ECG Error bar 95% confidence interval Blue bar - mean pre-test score; red bar - post-test score; green bar - retention post-test

The mean scores at baseline (pre-test), after two weeks of completion of the intervention (post-test), and after eight weeks of the completion of the intervention (retention post-test) according to the groups are given in Figure 2 and Figure 3 for normal and abnormal ECG, respectively.

In normal ECG, there was a significant main effect of teaching on the score: F(1,121)=5.56, p=0.02, η2=0.044. Average scores were significantly higher in the immersion group (mean 19.8, standard error (SE) 0.57) than in the traditional group (mean 17.9, SE 0.56). There was also a significant interaction effect between time and teaching method, F(2, 242)=5.20, p=0.006, η2=0.041, suggesting scores changed differently over time depending on the teaching program. There was a significant difference between immersion and traditional groups in the retention post-test mean scores (p<0.001) and no significant difference in the pre-test (p=0.281) and post-test (p=0.757) scores for normal ECG. Mean scores of pre-test, post-test, and retention post-test for both normal and abnormal ECG are summarized in (Table 2).

**Table 2:** Pre-intervention scores (pre-test), post-test score after two weeks of completion of intervention, and retention Post-test scores after two months of completion of intervention for immersion and traditional group Values are given as mean (SD)

Variables	Pre-test score	Post-test score	Retention post-test score	p-value
Normal ECG				
Immersion group	9.8 (8.4)	24.3 (5.5)	25.3 (5.6)	0.02
Traditional group	8.3 (7.5)	24.8 (6.3)	20.7 (6.9)	
p-value	0.281	0.757	<0.001	
Abnormal ECG				
Immersion group	7.0 (6.0)	23.5 (6.2)	19.2 (6.9)	0.001
Traditional group	8.3 (6.6)	17.7 (9.0)	13.3 (10.2)	
p-value	0.535	<0.001	0.001	

There were statistically significant changes for normal ECG in score over time for both the traditional group F(2, 122)=106.85, p<0.001) and immersion group F(2, 120)=127.97, p<0.001). The pairwise comparison indicates that for the traditional group, there were significant changes in scores from the pre- to post-intervention time points (p<0.001), from the pre-to-retention test (p<0.001), and from the post-intervention to retention test (p=0.001). Similarly, the pairwise comparison in the immersion group indicates that there was a significant change in score from the pre to post-intervention time points (p<0.001) and from pre-to-retention time (p<0.001) but not from post-to-retention time (p=0.855).

In abnormal ECG, there was a significant main effect of teaching on score F(1,121)=17.99, p<0.001, η2=0.129. Average scores were significantly higher in the immersion group (mean 16.6, SE 0.59) than in the traditional group (mean 13.6, SE 0.58). There was also a significant interaction effect between time and teaching method F(2, 242)=9.28, p<0.001, η2=0.071, suggesting scores changed differently over time depending on the teaching program. There is no statistically significant difference between immersion and traditional groups in the pre-test mean (p=0.535); however, a significant difference was observed in the post-test mean (p<0.001) and retention post-test mean (p=0.001) for abnormal ECG. 

There were statistically significant changes in score over time for both the traditional group F(2, 122)=18.76, p<0.001) and immersion group F(2, 120)=112.65, p<0.001). The pairwise comparison indicates that for the traditional group, there were significant changes in scores from the pre to the post-intervention time points (p<0.001), from the pre-to-retention test (p=0.004), and from the post-intervention to retention test (p=0.034). However, the pairwise comparison of the immersion group indicates that all the combinations and changes in score from the pre to post-intervention time points, from pre-to-retention time, and from post-to-retention time were significant (p<0.001).

## Discussion

The electrocardiogram (ECG) is one of the most important tools to diagnose cardiac abnormality, particularly arrhythmias and myocardial infarctions. ECG interpretation skills require clinical knowledge and regular interpretation of ECG. Interpretation of normal ECG is taught in the first year of medical school in a department of physiology. The teaching method mainly involves didactic lecturers with hands-on training in practical classes. The first-year medical students are not well versed in the clinical conditions; hence their skill to interpret ECG is limited. In the subsequent years of their academic program, students acquire the skill during their clinical postings. A study done at a medical school in New Zealand among final-year medical students and residents did not achieve the required competency to interpret different clinical conditions in ECG [1]. This trend was seen across the globe in various other studies [4-7]. A meta-analysis by Cook et al. found that physicians at all levels had deficiencies in interpreting ECG [8].

In this randomized controlled study, we used virtual reality as a teaching-learning tool to train the students to interpret ECG. During an extensive literature search, no study was found using virtual reality as a teaching tool to train students in interpreting ECG. To understand the students' baseline knowledge, we asked whether they attended the basics of ECG classes during their first year of medical school. There was no difference between the groups concerning gender and attending the class in first-year medical school. The baseline pretest for both normal and abnormal ECG showed no difference among the groups (p>0.05).

The post-test to assess the interpretation skill of normal ECG among both groups was not significant, with a p-value of 0.757. A study done at one of the medical schools in Poland also demonstrated that medical students have a good level of competency in interpreting normal ECG, but they lack the desired competency level to interpret abnormal ECG [5]. Another study by Jablonover et al. and Al Mousa et al. also showed a similar result among medical students [6,7].

The post-test for interpretation of abnormal ECG showed statistically significant differences among the groups with a p-value of <0.001. A study using blended learning showed results similar to our study. In our study, the two groups were trained with two distinct teaching methods, but in the study, using blended learning by Viljoen et al. [3], the immersion group had the opportunity to attend didactic lectures along with web-based tutorials. The mean score in the blended learning group increased 2.4-fold compared to 1.6-fold in the didactic group (p<0.001). The post-test for acquisition of knowledge was done immediately after the intervention compared to our study, where the post-test was done after two weeks of intervention.

The web-based tutorial also has a positive impact on the ECG interpretation skills of medical students. A study by Pourmand et al. and Rolskov et al. concluded that web-based ECG tutorials can be an effective method to teach ECG to students; however, students may not retain the knowledge for a longer duration [9,10]. A computer-based simulation for training students on ECG interpretation skills showed a similar result with a p-value of 0.000 compared to the traditional method [11,12]. Another study comparing e-learning and near-peer teaching showed increased confidence in interpreting ECG, although the near-peer method was slightly better than e-learning in the study [13].

Retention of knowledge for interpretation of ECG is a challenge for the students. One of the main reasons for students having low confidence in interpreting ECG is the didactic method used for teaching. This was demonstrated by Amini et al. [2] in a study where they recommended that regular training and self-assessment are required to improve the confidence level of the students. In our study, the immersion group showed significant improvement in the retention post-test score for both normal and abnormal ECG (p<0.001). The only other study by Viljoen et al., using blended learning, showed better retention of knowledge [3].

Case-based training of ECG is another method that has shown a positive effect on acquiring knowledge on the interpretation of ECG [6,14]. A recent meta-analysis by Ardekani et al. [15] concluded that training students to interpret ECG should utilize novel and creative methods. The study also suggested the use of self-directed learning, peer teaching, or computer-based ECG training may be beneficial for students.

This study is one of its kind, using virtual reality to train the students on not only acquiring knowledge but also retaining the knowledge of interpreting ECG. The virtual reality training was more intense, and students had the opportunity to do the module multiple times; this could have been one of the factors for significant differences among the groups. The time interval between training and assessing the retention skill may not be enough in this study to conclude its effectiveness as a teaching-learning tool.

## Conclusions

This randomized controlled study using virtual reality demonstrated significant improvement in acquiring and retaining knowledge of interpreting both normal and abnormal ECG compared to the traditional teaching method. Virtual reality can mimic the real environment where students can interact with virtual patients and learn to interpret ECG of various clinical scenarios. It can be used as self-directed learning, and students can learn at their own pace, giving a holistic approach to the learning process.
